# Supplementary material for: Frequency-dependent selection of neoantigens fosters tumor immune escape and predicts immunotherapy response
Source: Commun Biol. 2024 Jun 25;7:770. doi: 10.1038/s42003-024-06460-7 (PMC11199503; doi:10.1038/s42003-024-06460-7)
Supplement: Supplementary file 2 — Supplementary Information [file 42003_2024_6460_MOESM2_ESM.pdf]

1 Supplementary Figures

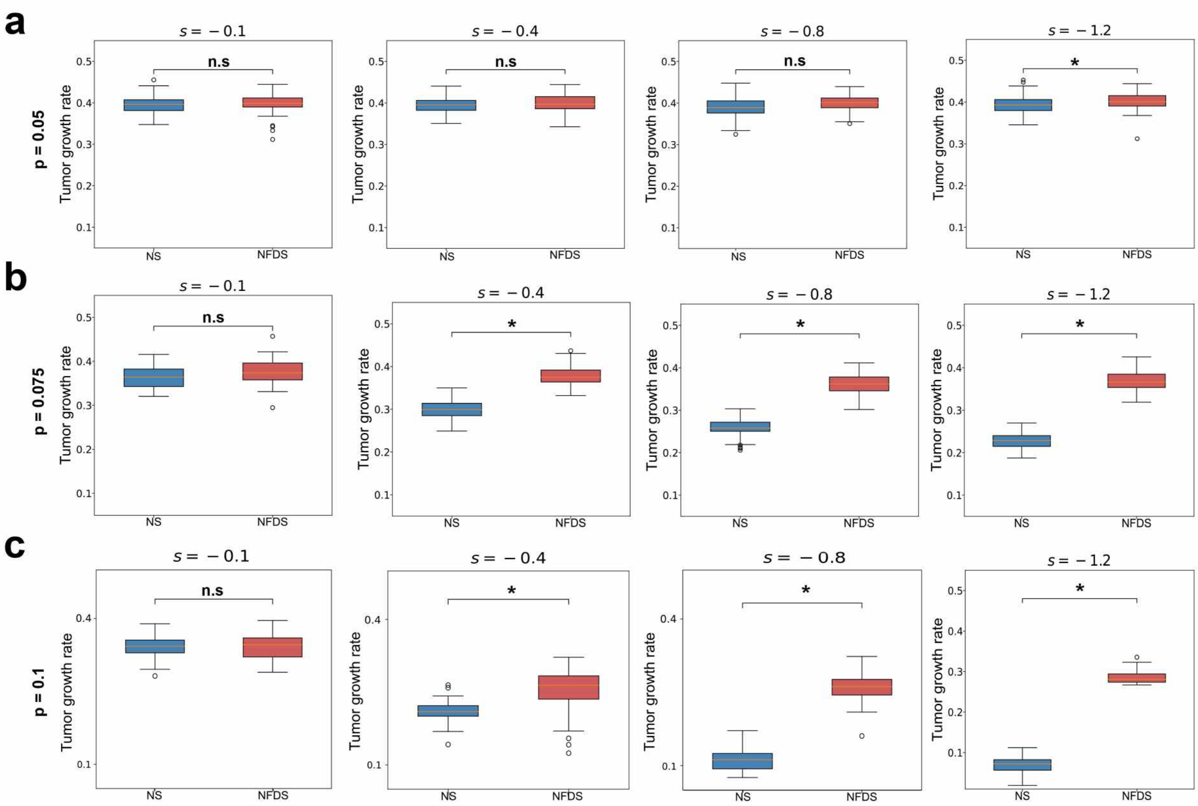

Supplementary Fig. 1. Tumor growth rates with varying neoantigen acquisition rates and negative selection intensities. a-c, Box plots showing tumor growth rates in 50 simulated tumors under NS and NFDS with neoantigen acquisition rate equals 0.05 (**a**), 0.075 (**b**) and 0.1 (**c**). * represents a p value less than 0.05 and $n.s$ represents no significant difference, one-sided Wilcoxon rank-sum test.

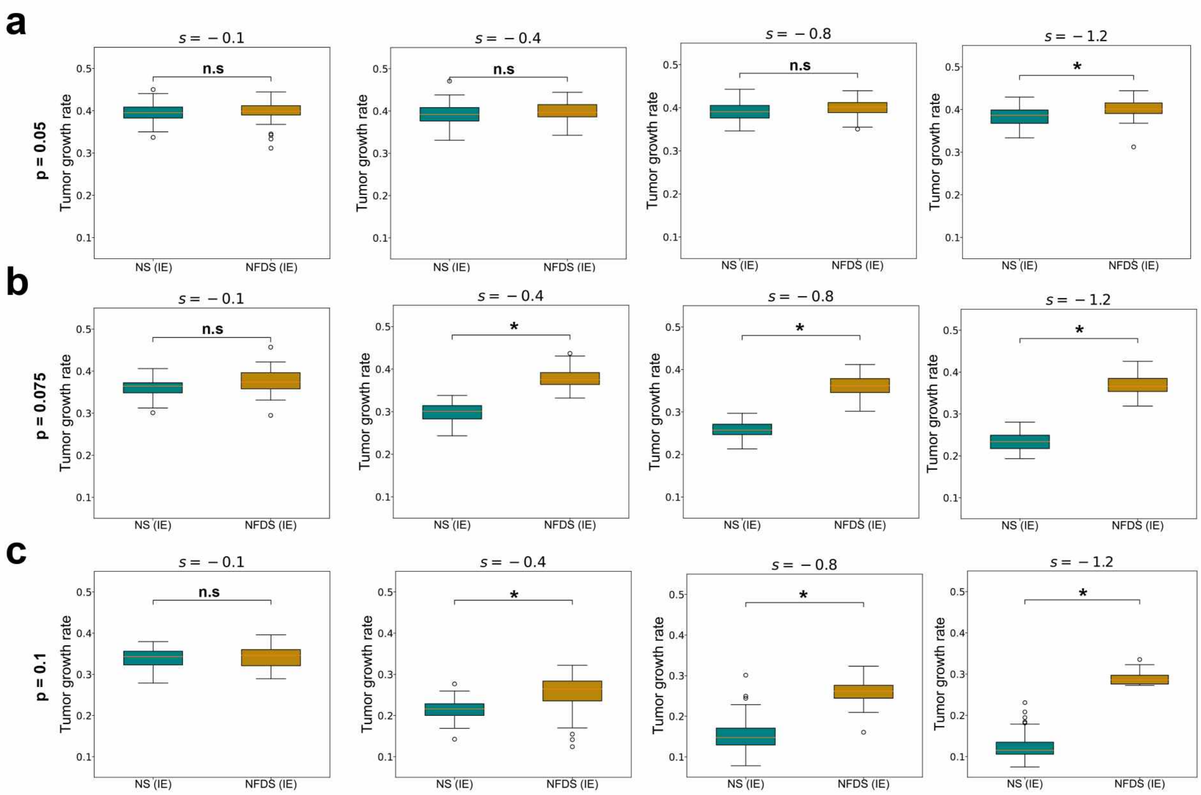

Supplementary Fig. 2. Tumor growth rates with varying neoantigen acquisition rates and negative selection intensities under immune escape (IE). a-c, Box plots showing tumor growth rates in 50 simulated tumors under NS (IE) and NFDS (IE) with neoantigen acquisition rate equals 0.05 (**a**), 0.075 (**b**) and 0.1 (**c**). * represents a p value less than 0.05 and $n.s$ represents no significant difference, one-sided Wilcoxon rank-sum test.

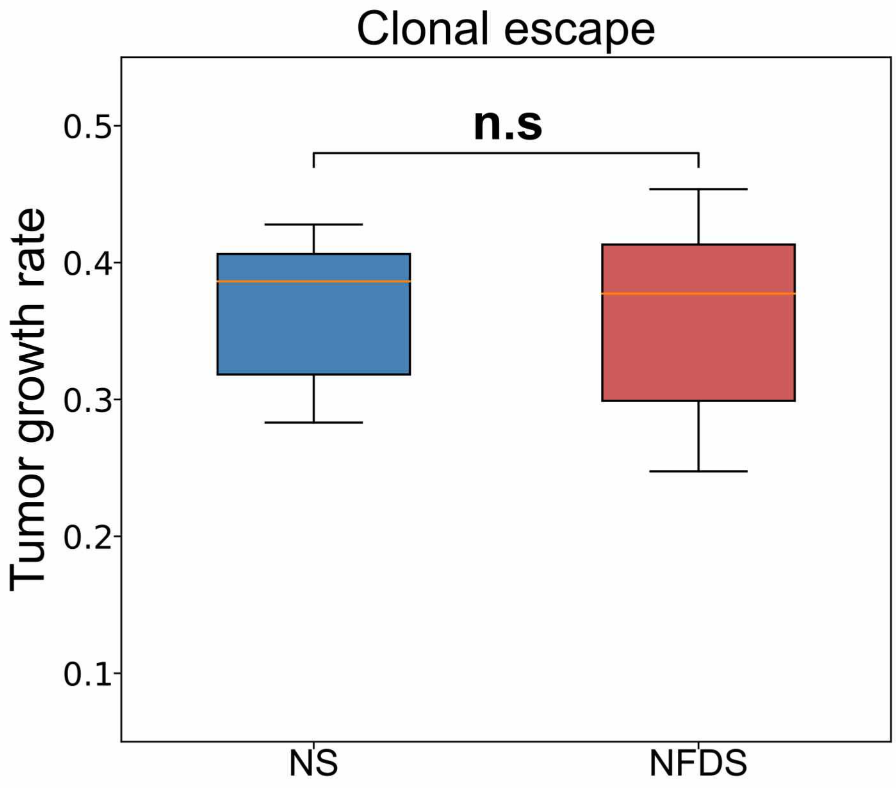

Supplementary Fig. 3. Tumor growth rates with clonal escape. Box plots showing tumor growth rates in 50 simulated tumors under NS (IE) and NFDS (IE) with $p_e = 1$ to simulated clonal escape. $n.s$ represents no significant difference, one-sided Wilcoxon rank-sum test.

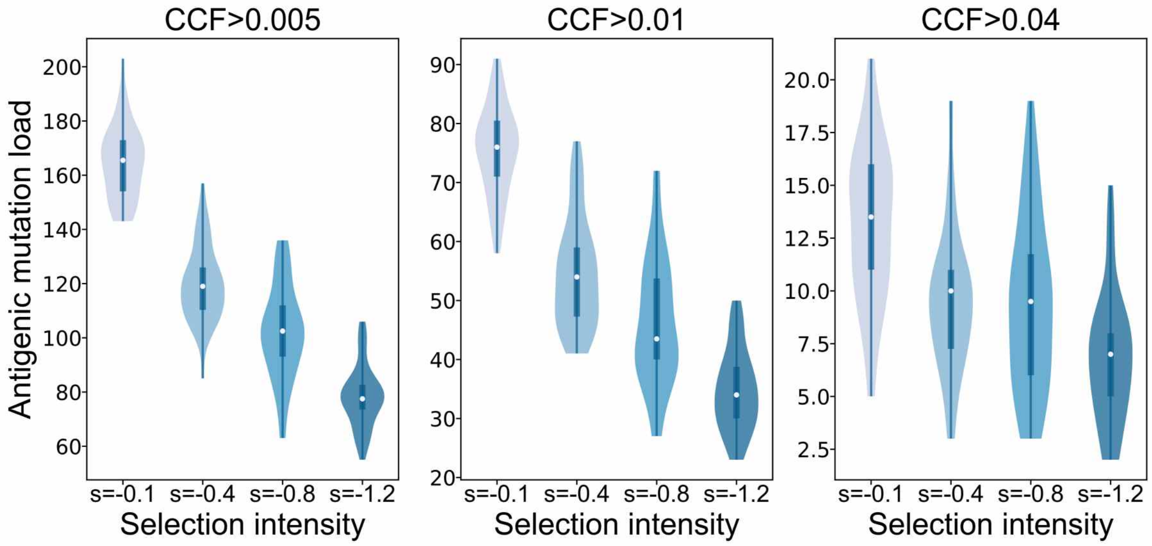

Supplementary Fig. 4. Neoantigen loads with different CCF cutoffs. Number of neoantigens accumulated in simulated tumors undergoing NS at varying selective intensities (each with 50 simulations). Cutoffs of neoantigen clonality CCF $>$ 0.005, 0.01 and 0.04 are used, respectively. Violin plots show median, quartiles and range (whiskers).

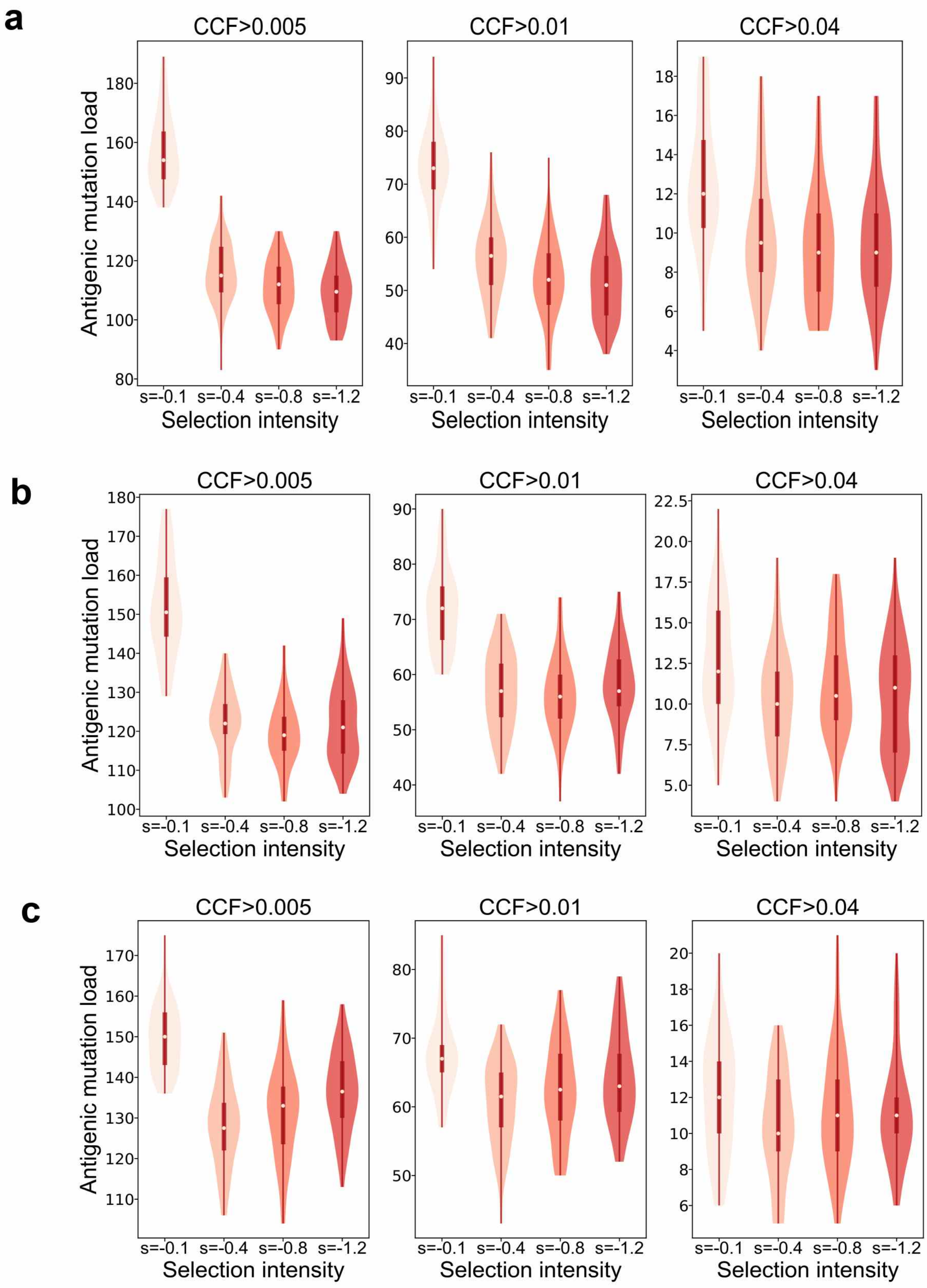

Supplementary Fig. 5. Neoantigen loads with different cell immunogenicity cutoffs. a-c, Number of neoantigens accumulated in simulated tumors at varying selective intensities (each with 50 simulations). Simulated tumors undergoing NFDS with cell immunogenicity threshold (c_1) equals 0.3 (**a**), 0.5 (**b**) and 0.7 (**c**) are shown. Neoantigens with CCF $>$ 0.005,0.01,0.04 are used, respectively. Violin plots show median, quartiles and range (whiskers).

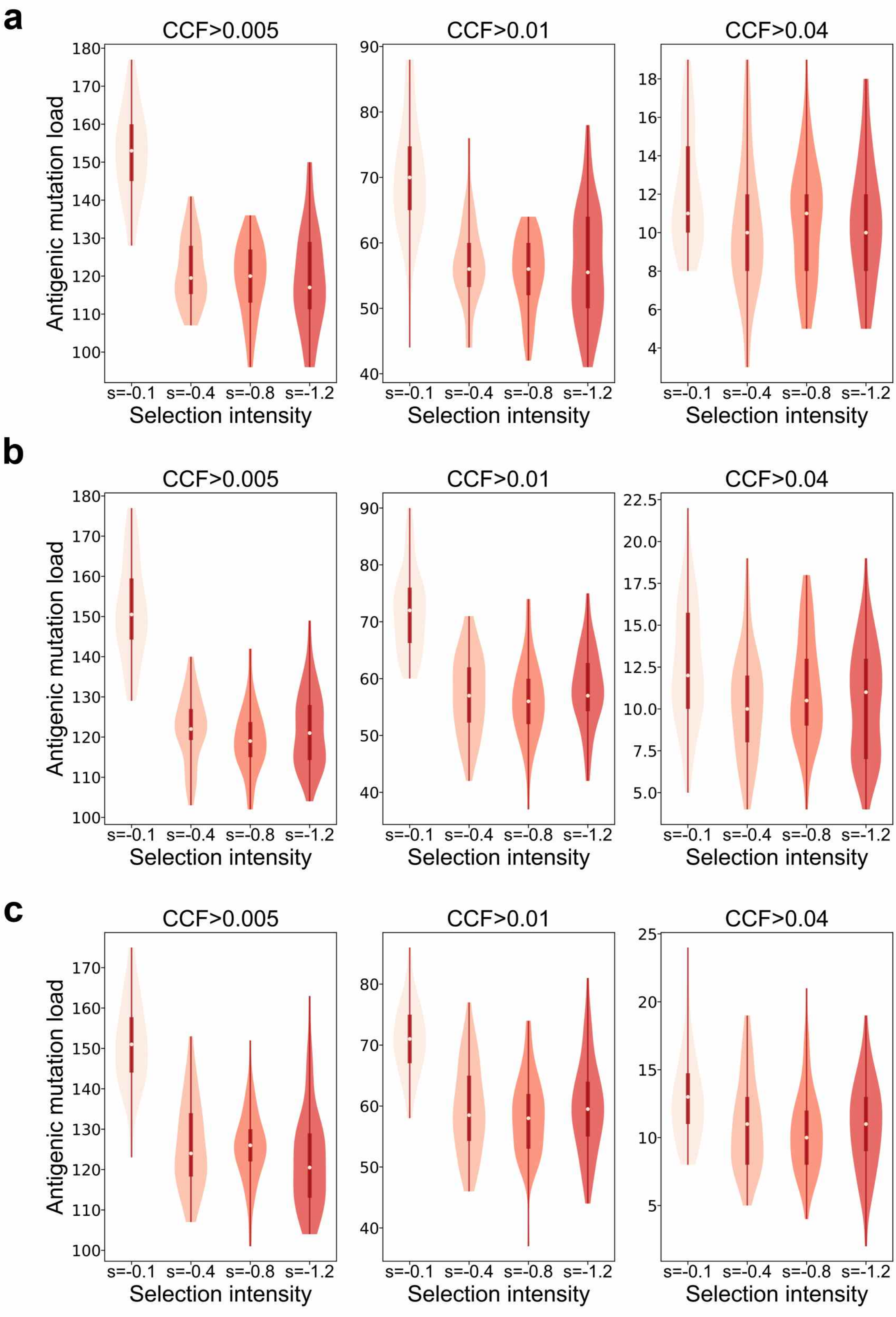

Supplementary Fig. 6. Neoantigen loads with different tumor immunogenicity cutoffs. a-c, Number of neoantigens accumulated in simulated tumors at varying selective intensities (each with 50 simulations). Simulated tumors undergoing NFDS with cell immunogenicity threshold (c_2) equals 0.3 (**a**), 0.5 (**b**) and 0.7 (**c**) are shown. Neoantigens with CCF $>$ 0.005,0.01,0.04 are used, respectively. violin plots show median, quartiles and range (whiskers).

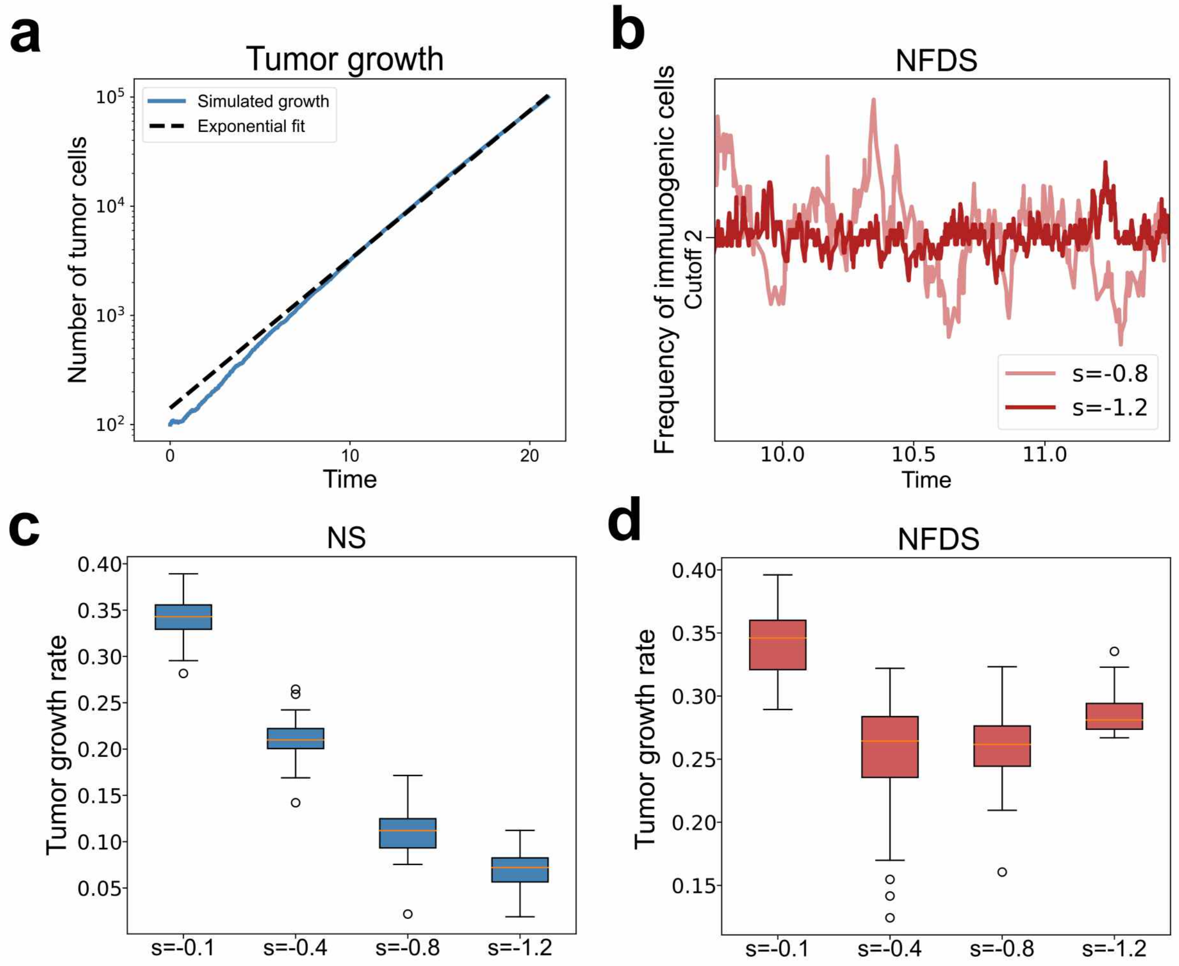

Supplementary Fig. 7. Tumor growth rates under NS and NFDS, respectively. a, Exponential fitting of simulated tumor growth. **b,** Frequency curves of immunogenic cells of two simulated tumors under negative frequency-dependent selection (NFDS) with selective intensity $s = -0.8$ and -1.2, respectively. **c-d,** Tumor growth rate at varying selective intensities in simulated tumors (n = 50) undergoing NS and NFDS, respectively. Box plots show median, quartiles (boxes) and range (whiskers).

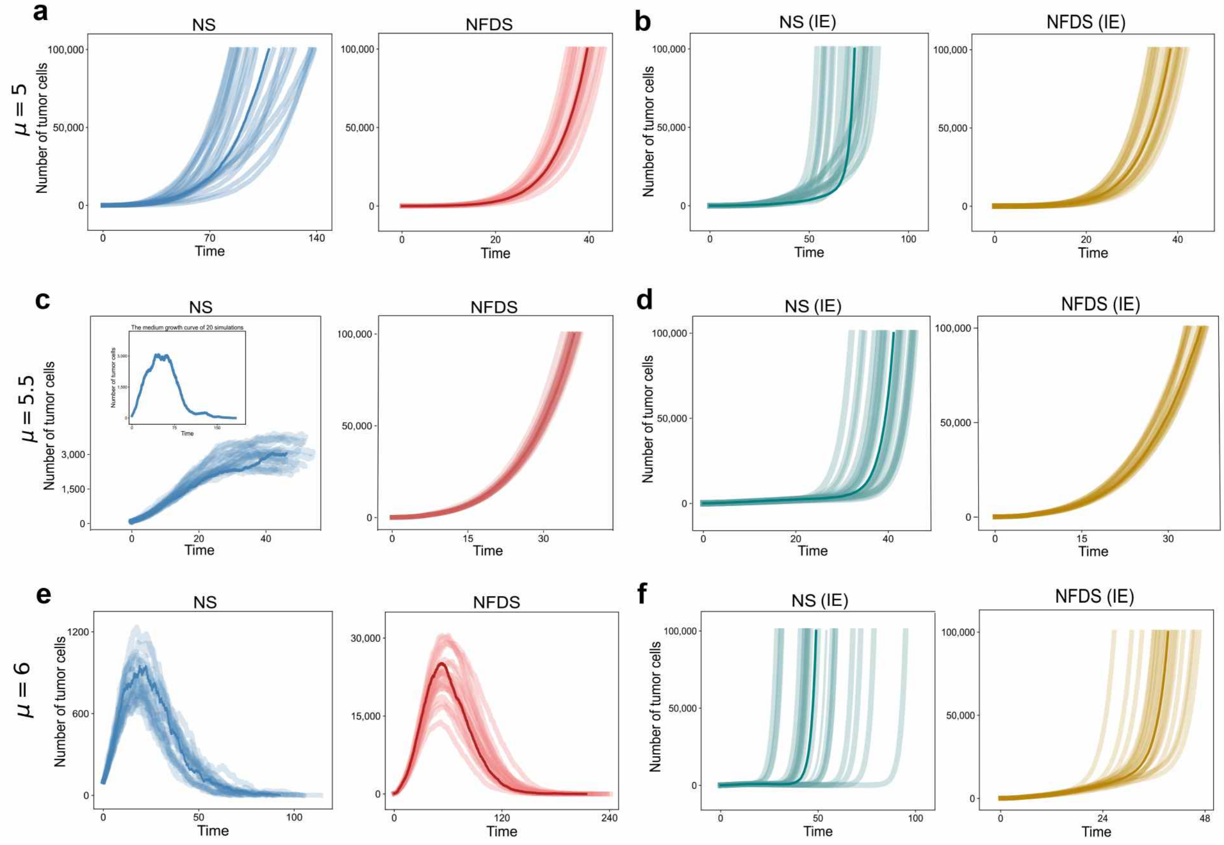

Supplementary Fig. 8. Tumor growth curves with different mutation rates. **a,** Growth curves of 20 simulated tumors under NS and NFDS, respectively. **b,** Growth curves of 20 simulated tumors under NS (IE) and NFDS (IE), respectively. Here mutation rate $\mu = 5$ and selection coefficient $s = -0.8$ are used. **c,** Growth curves of 20 simulated tumors under NS and NFDS, respectively. **d,** Growth curves of 20 simulated tumors under NS (IE) and NFDS (IE), respectively. Here mutation rate $\mu = 5.5$ and selection coefficient $s = -0.8$ are used. **e,** Growth curves of 20 simulated tumors under NS and NFDS, respectively. **f,** Growth curves of 20 simulated tumors under NS (IE) and NFDS (IE), respectively. Here mutation rate $\mu = 6$ and selection coefficient $s = -0.8$ are used.

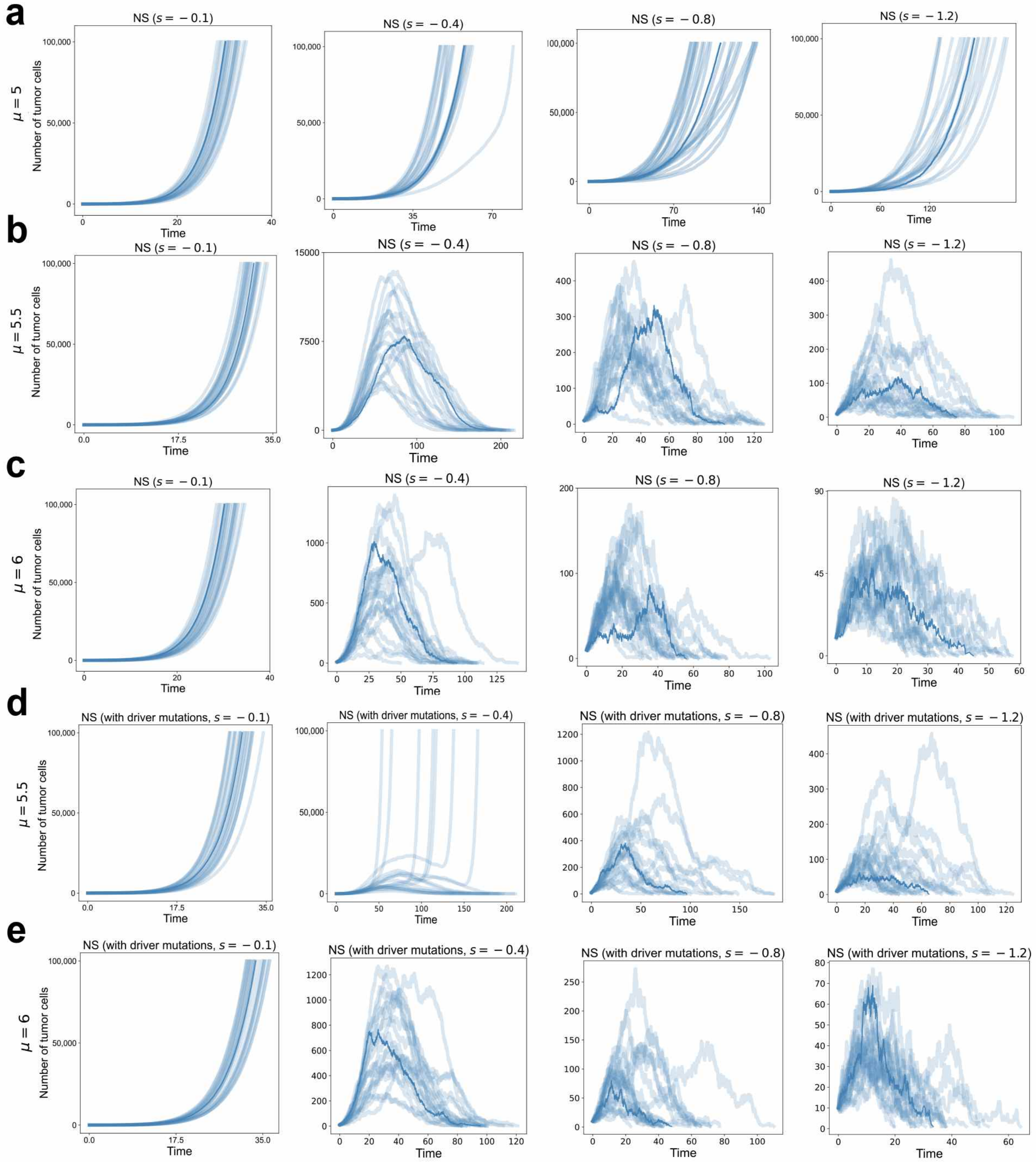

Supplementary Fig. 9. Tumor growth curves of NS with diverse parameters. a-c, Growth curves of 20 simulated NS tumors with mutation rate set to $\mu = 5$ (**a**), $\mu = 5.5$ (**b**) and $\mu = 6$ (**c**), respectively. **d-e,** Growth curves of 20 simulated NS tumors with the rate of driver events set to 10^{-6} under mutation rates of $\mu = 5.5$ (**d**) and $\mu = 6$ (**e**), respectively.

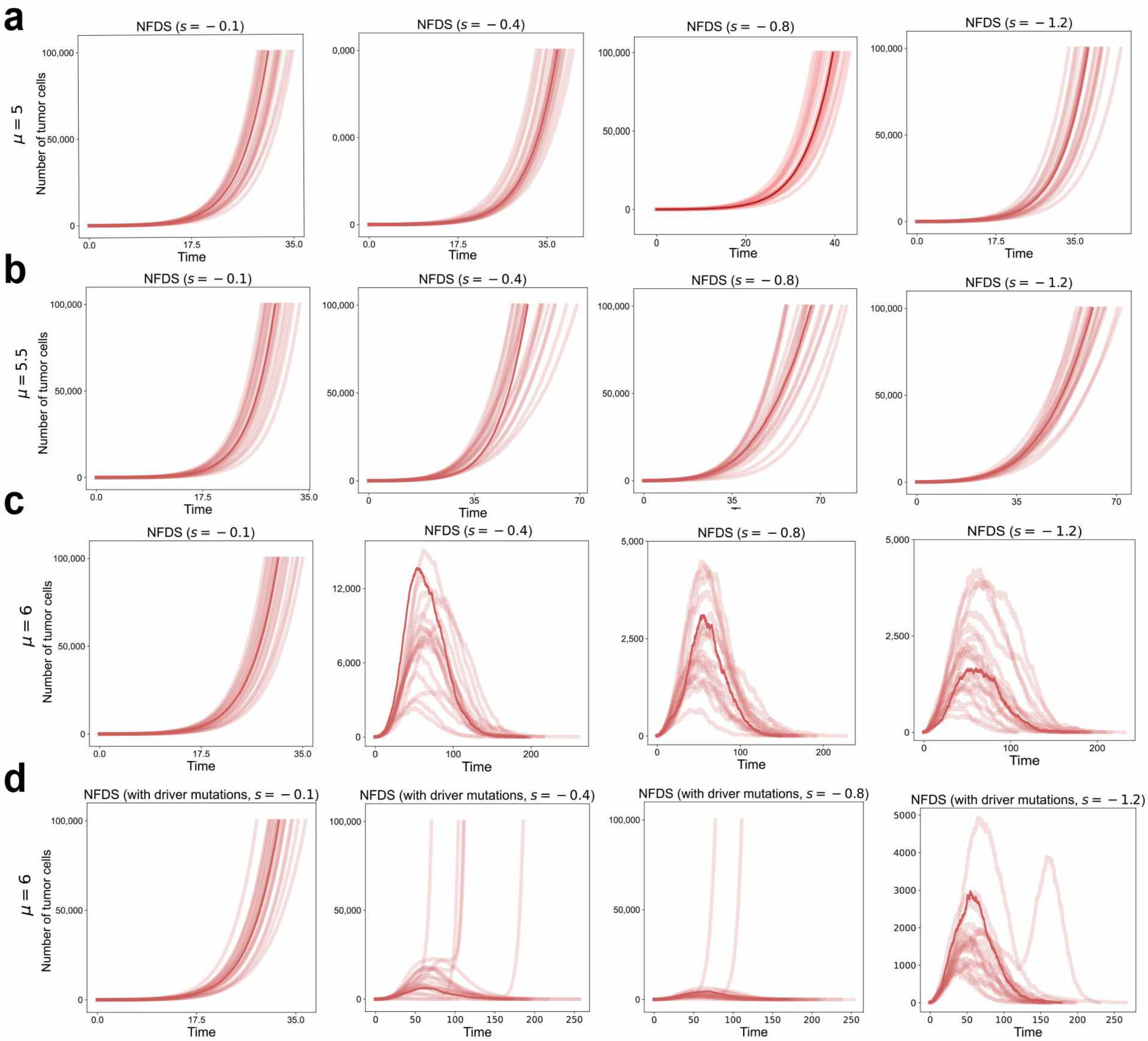

Supplementary Fig. 10. Tumor growth curves of NFDS with diverse parameters. a-c, Growth curves of 20 simulated NFDS tumors with mutation rate set to $\mu = 5$ (**a**), $\mu = 5.5$ (**b**) and $\mu = 6$ (**c**), respectively. **d,** Growth curves of 20 simulated NFDS tumors with the rate of driver events set to 10^{-6} under mutation rate of $\mu = 6$.

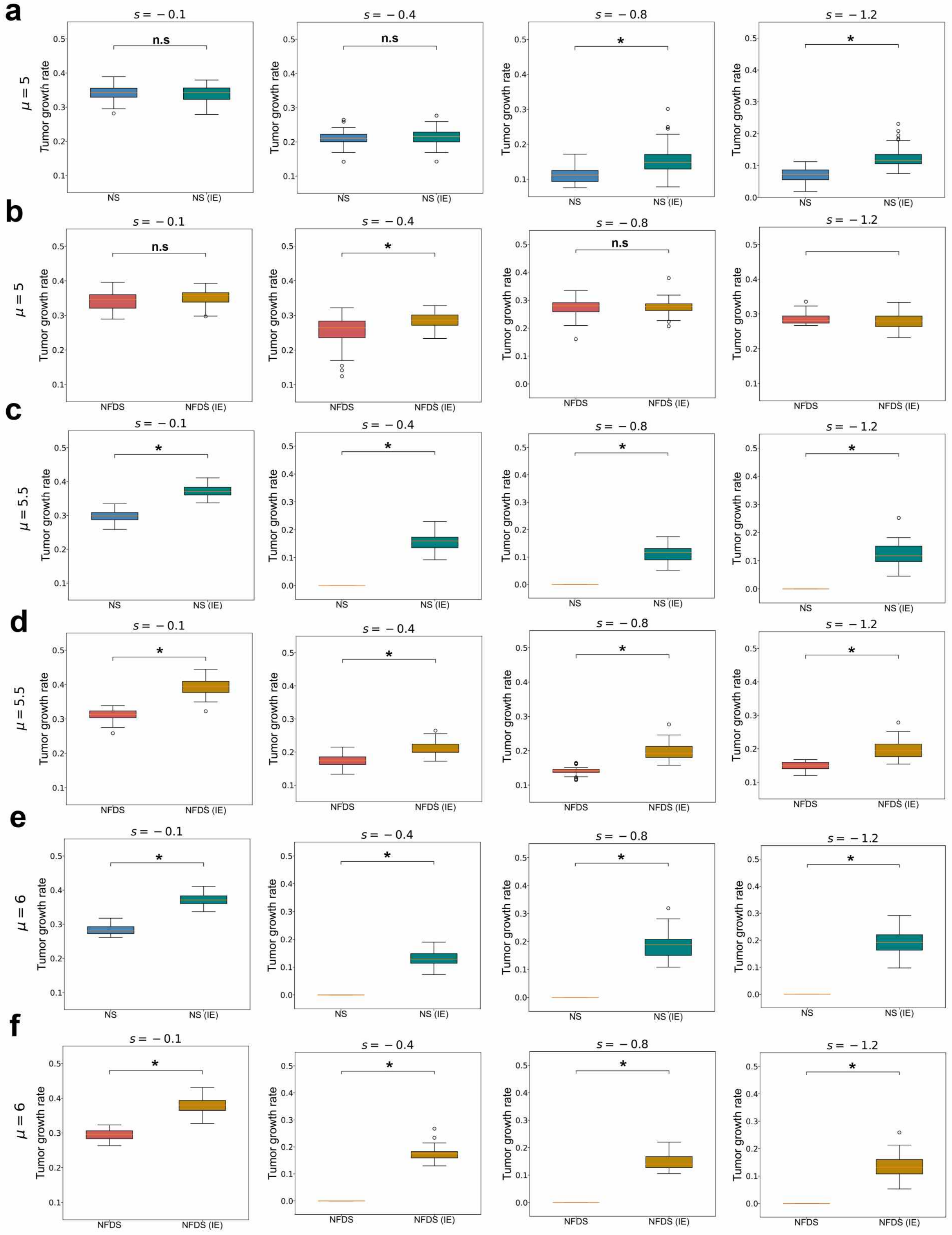

Supplementary Fig. 11. Effect of IE on tumor growth rate. a-b, Box plots showing tumor growth rates in 50 simulated tumors under NS (**a**) and NFDS (**b**) at varying s with mutation rate of $\mu = 5$. **c-d,** Box plots showing tumor growth rates in 50 simulated tumors under NS (**c**) and NFDS (**d**) at varying s with mutation rate of $\mu = 5.5$. **e-f,** Box plots showing tumor growth rates in 50 simulated tumors under NS (**e**) and NFDS (**f**) at varying s with mutation rate of $\mu = 6$.

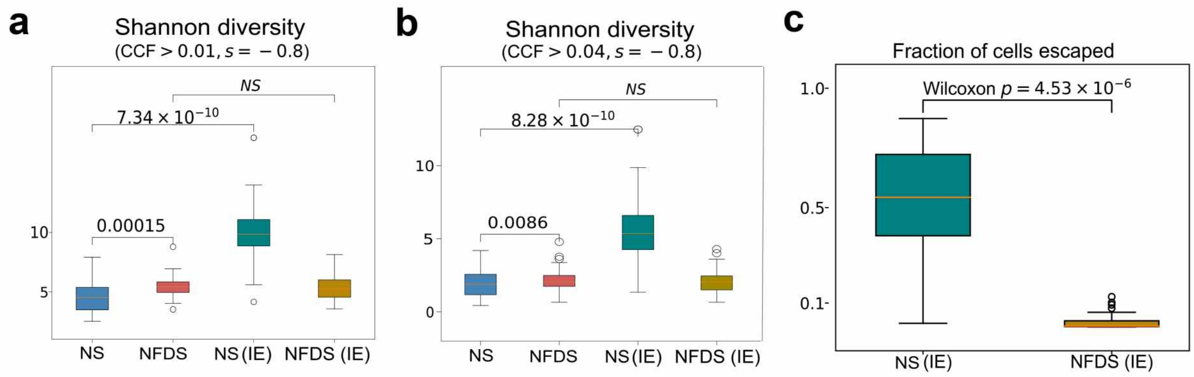

Supplementary Fig. 12. Shannon diversity and fraction of immune-escaped cells. a-b, Box plots showing the antigenic mutations accumulated in 50 simulated tumors with cancer cell fraction (CCF) $>$ 0.01 (**a**) and CCF $>$ 0.04 (**b**), respectively. The Shannon diversity of antigenic mutations from 50 simulated tumors under four evolutionary scenarios are shown. **c,** Box plots of fractions of cells with escaped mutations in 50 simulated tumors under NS and NFDS with subclonal immune escape (IE), respectively. Box plots show median, quartiles (boxes) and range (whiskers). p values, one-sided Wilcoxon rank-sum test.

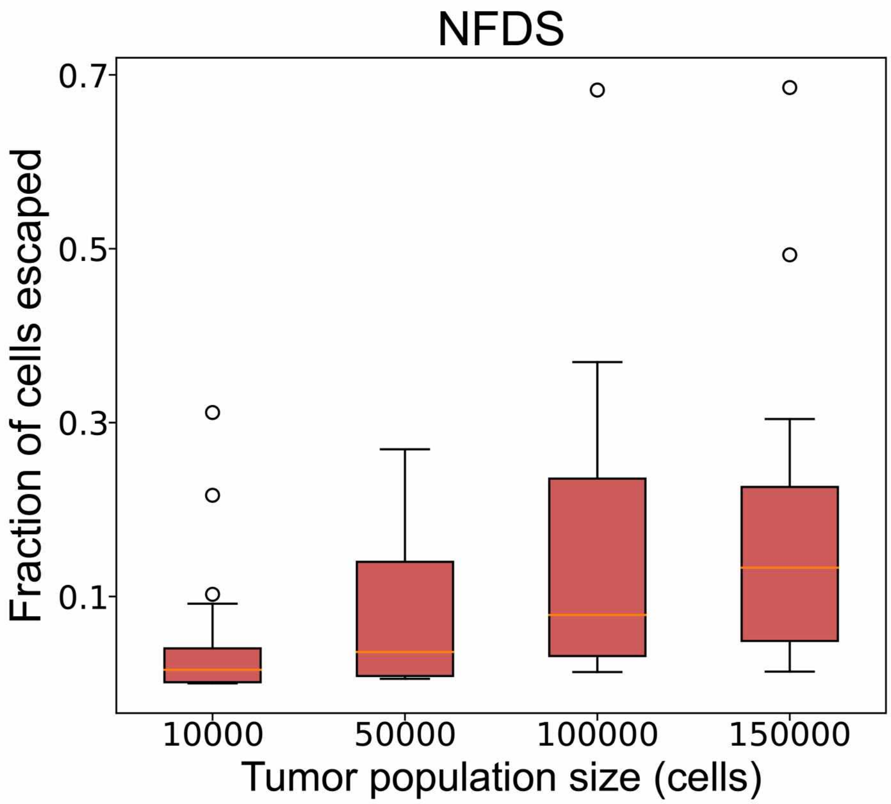

Supplementary Fig. 13. Prevalence of escaped cells. Box plots showing fractions of escaped cells in NFDS tumors with varying predefined population sizes.

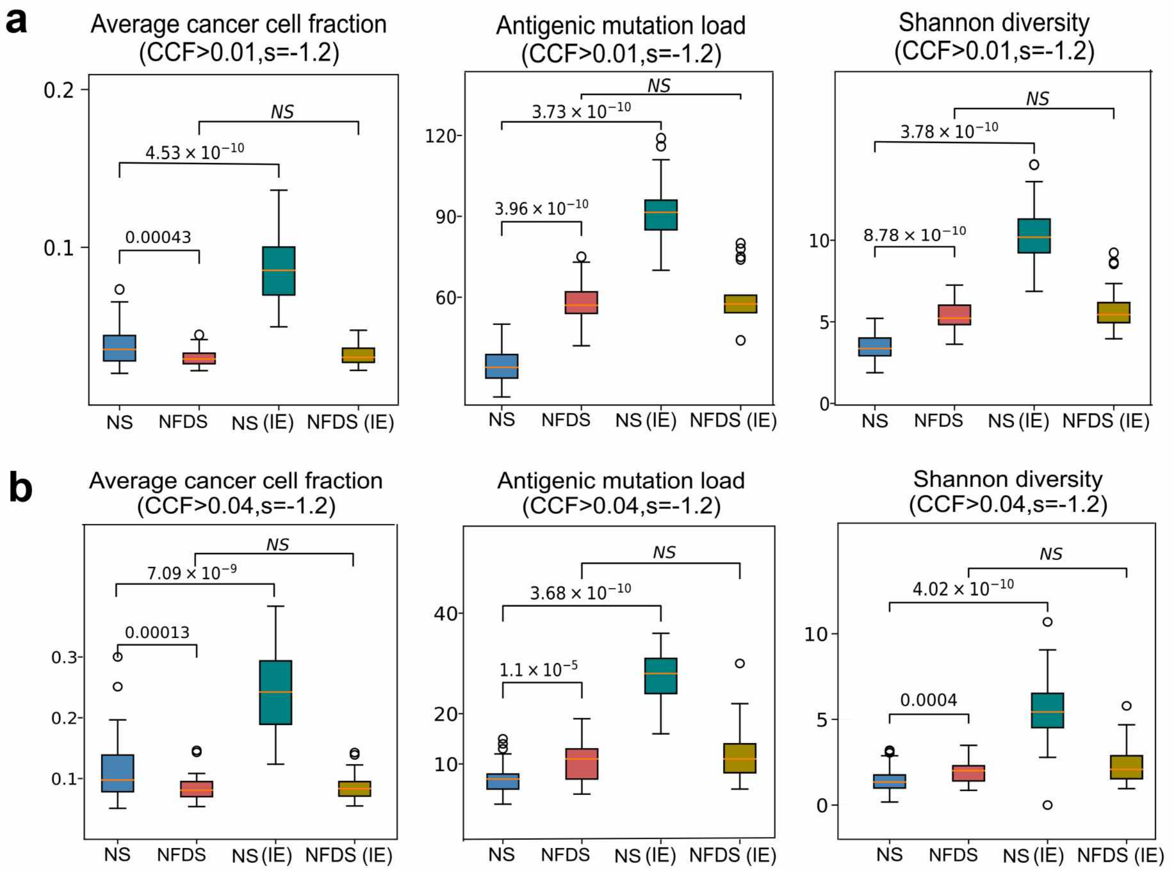

Supplementary Fig. 14. Cancer cell fraction (CCF), antigenic mutation load and Shannon diversity. a-b, Box plots showing the antigenic mutations accumulated in 50 simulated tumors with cancer cell fraction (CCF) > 0.01 (**a**) and CCF > 0.04 (**b**), respectively. The average CCF, mutation load and Shannon diversity of antigenic mutations from 50 simulated tumors under four evolutionary scenarios are shown. Box plots show median, quartiles (boxes) and range (whiskers). p values, one-sided Wilcoxon rank-sum test.

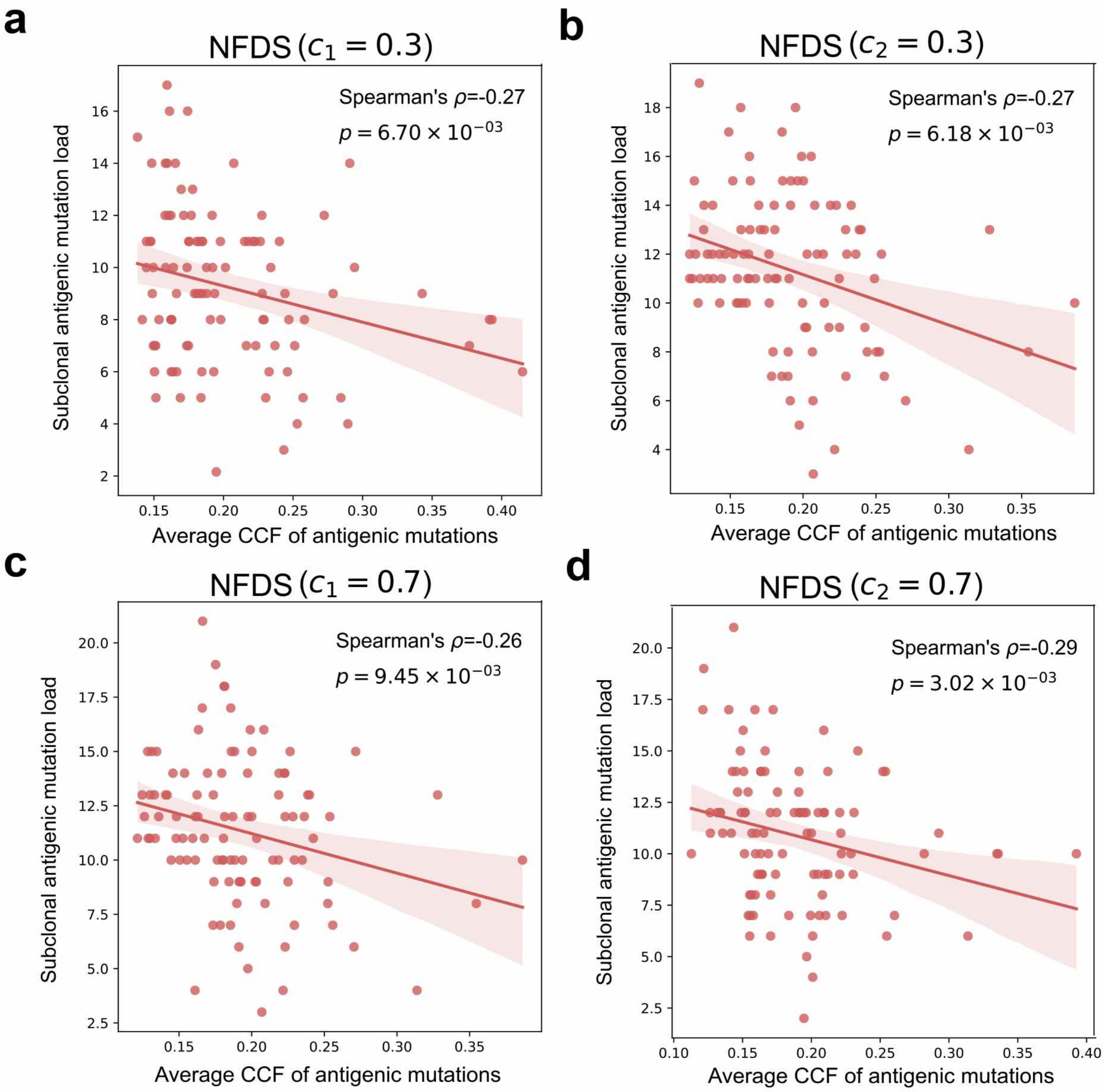

Supplementary Fig. 15. Correlation analysis between average CCF and subclonal antigenic mutation load under different immunogenicity cutoffs. a-b, Correlation analysis between average CCF and subclonal antigenic mutation load of 100 simulated tumors undergoing NFDS (CCF > 0.1) with cell immunogenicity cutoffs (c_1) equals 0.3 (**a**) and 0.7 (**b**), respectively. **c-d,** Correlation analysis between average CCF and subclonal antigenic mutation load of 100 simulated tumors undergoing NFDS (CCF > 0.1) with tumor immunogenicity cutoffs (c_2) equals 0.3 (**c**) and 0.7 (**d**), respectively. The line indicates the linear regression and the shading indicates the 95% CI of the regression.

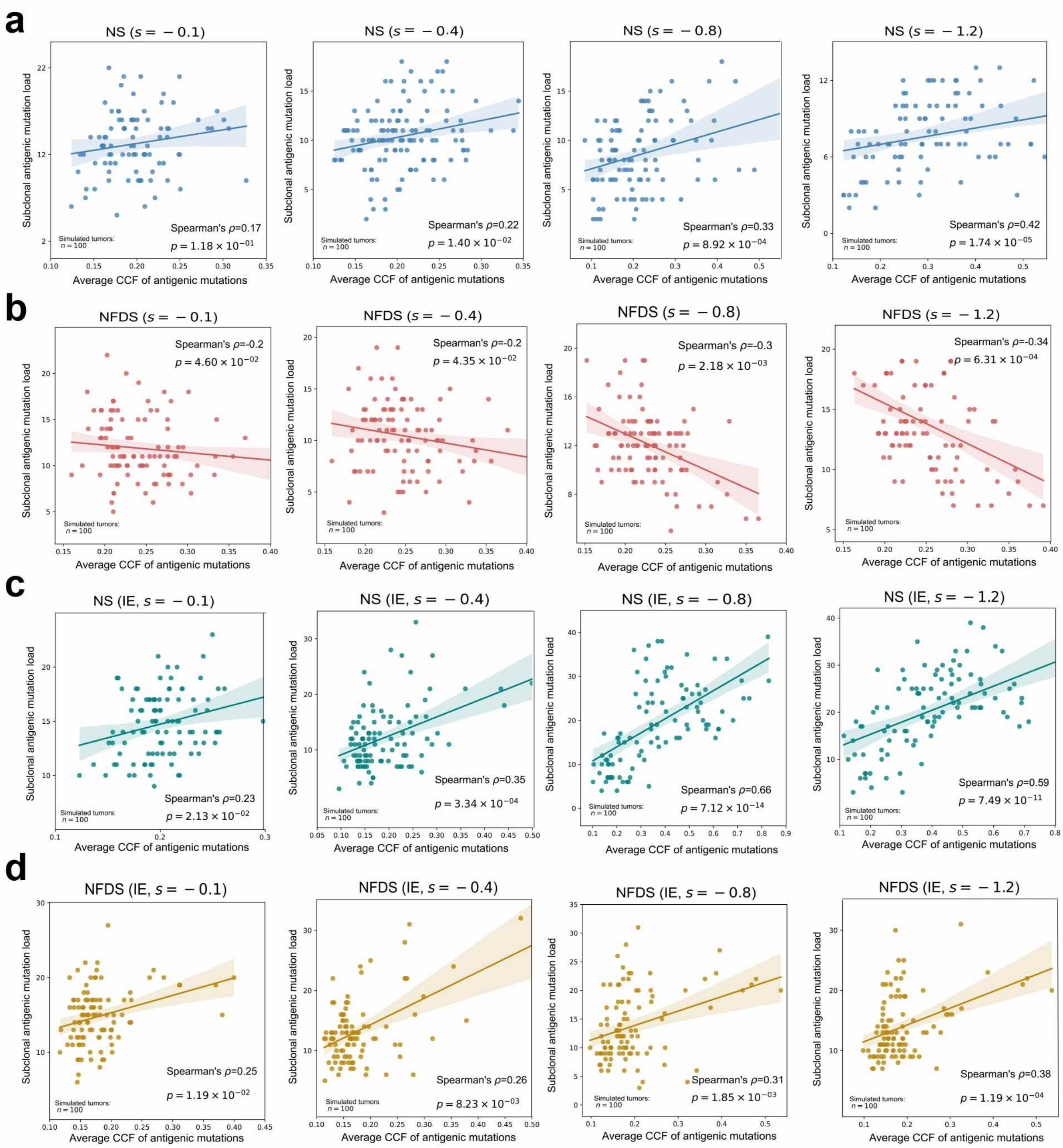

Supplementary Fig. 16. Correlation analysis between average CCF and subclonal antigenic mutation load. a-b, Correlation analysis between average CCF and subclonal antigenic mutation load of 100 simulated tumors undergoing NS (**a**) and NFDS (**b**) at varying s (CCF $>$ 0.1). **c-d,** Correlation analysis between average CCF and subclonal antigenic mutation load of 100 simulated tumors undergoing NS (IE) (**c**) and NFDS (IE) (**d**) at varying s (CCF $>$ 0.1). The line indicates the linear regression and the shading indicates the 95% CI of the regression.

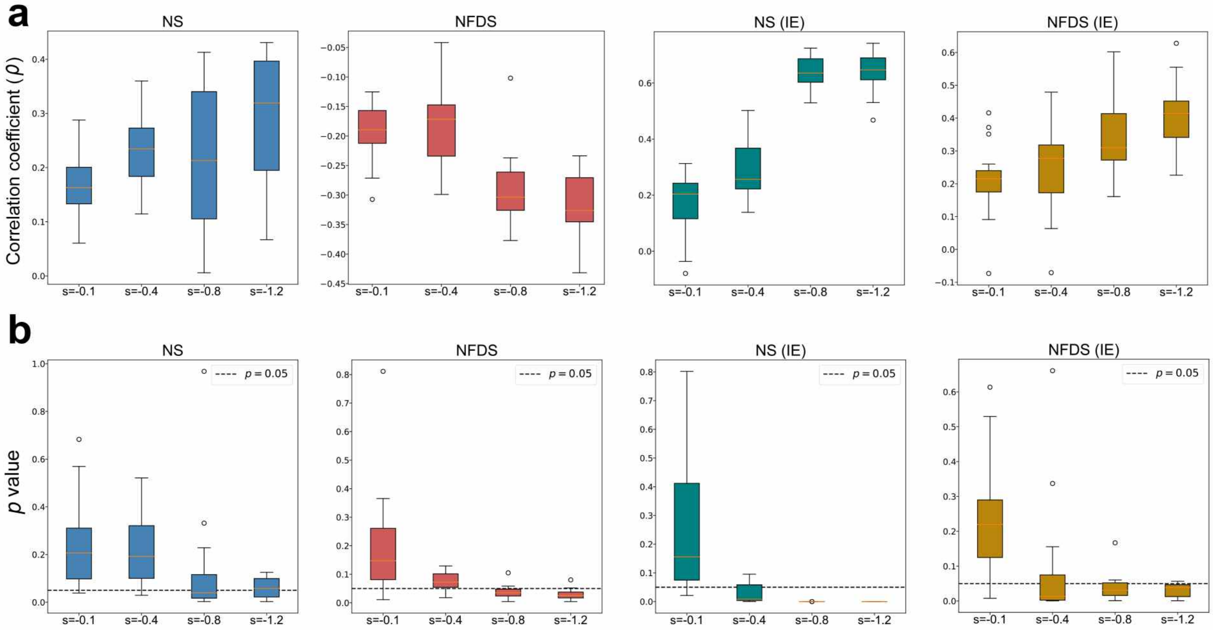

Supplementary Fig. 17. ρ and p distributions of correlation analysis. a, Box plots showing ρ values of correlation analysis between average CCF and antigenic mutation load under NS, NFDS, NS (IE) and NFDS (IE). **b,** Box plots showing p values of correlation analysis between average CCF and antigenic mutation load under NS, NFDS, NS (IE) and NFDS (IE). Box plots show median, quartiles (boxes) and range (whiskers).

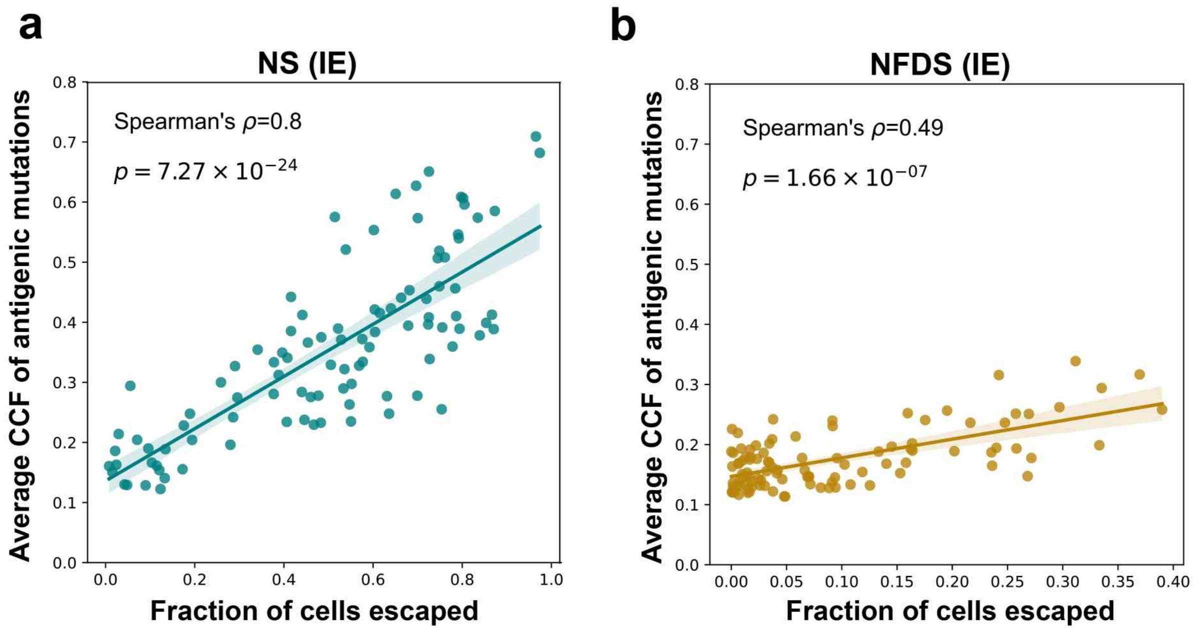

Supplementary Fig. 18. Correlation analysis between average CCF of neoantigens and fraction of immune-escaped cells. a-b, NS (**a**) and NFDS (**b**) with subclonal immune escape (immune escape probability $p_e = 10^{-4}$, CCF $>$ 0.1), respectively. The line indicates the linear regression and the shading indicates the 95% CI of the regression.

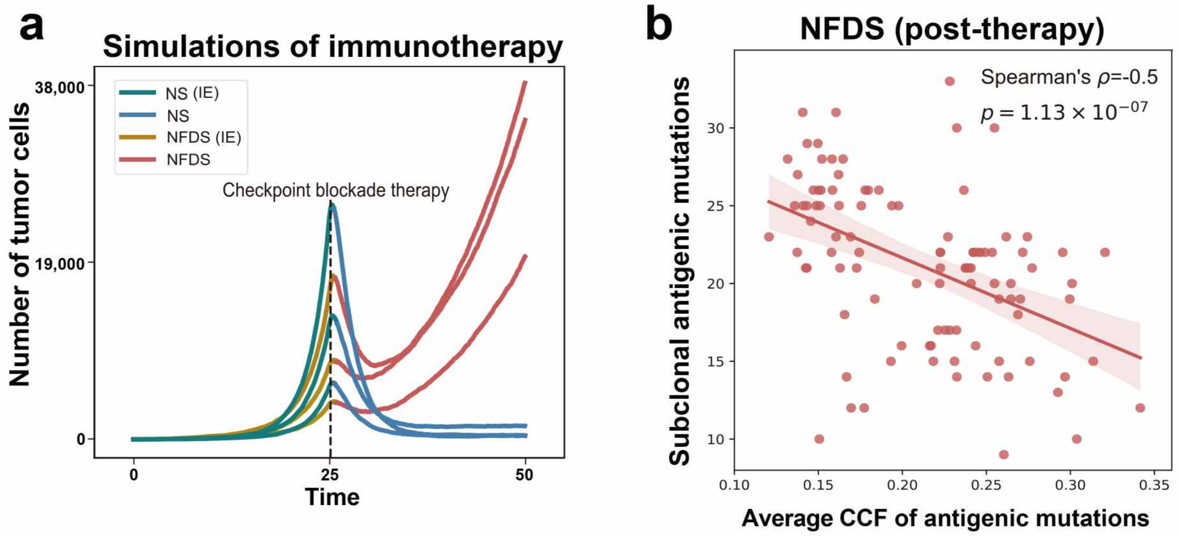

Supplementary Fig. 19. Response to immunotherapy and post-therapy features. a, Growth curves of 20 simulated tumors under NS and NFDS, respectively. **b,** Correlation analysis between average CCF and subclonal antigenic mutation load of ICB-treated *virtual* patients (n = 100) who underwent NFDS. Only neoantigens with CCF > 0.1 are used. The line indicates the linear regression and the shading indicates the 95% CI of the regression.

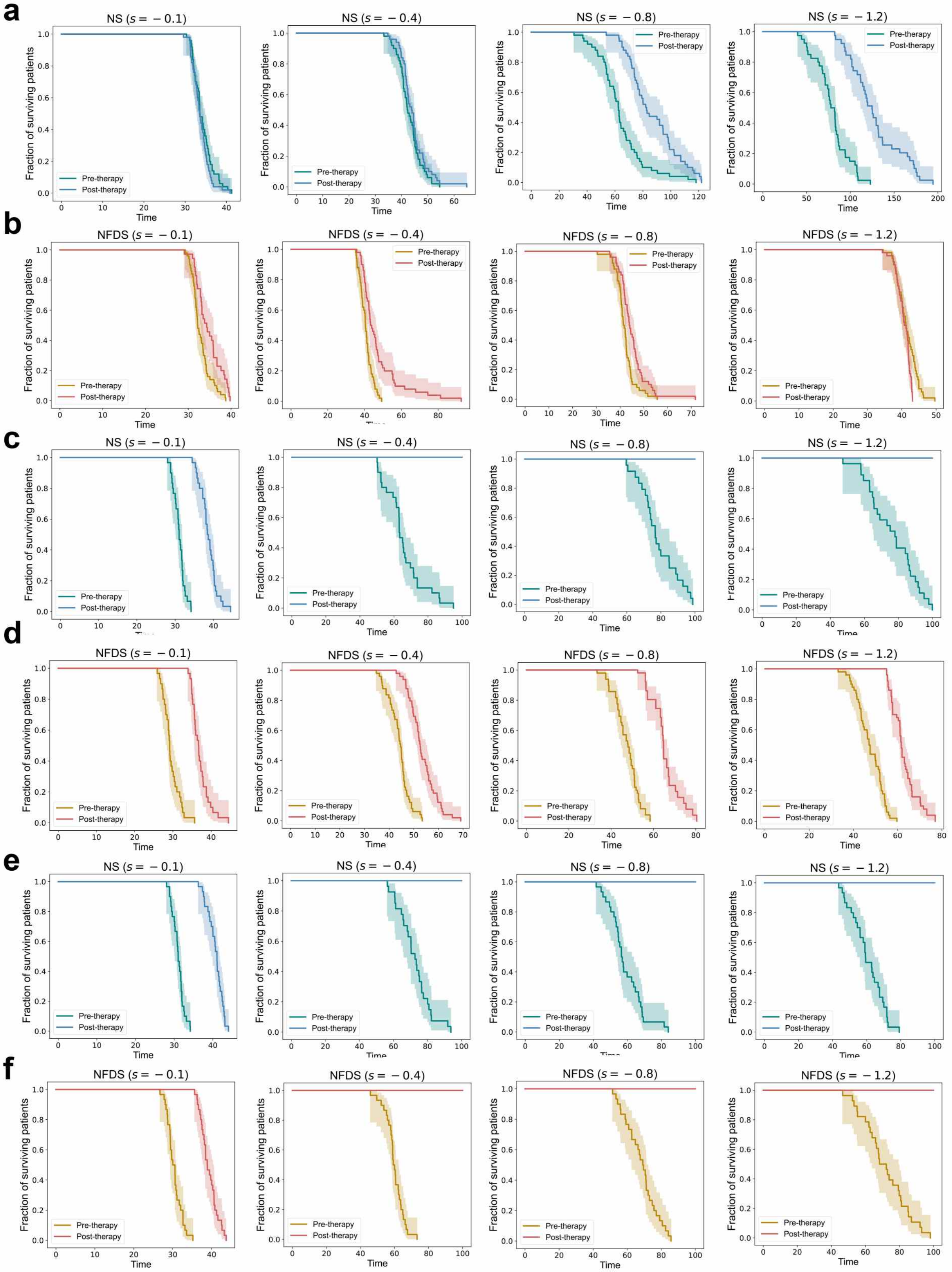

Supplementary Fig. 20. Survival analysis representing the benefit from ICB. a-b, Kaplan-Meier curves of 50 *virtual* patients that receive immunotherapy under NS (**a**) and NFDS (**b**) with mutation rate of $\mu = 5$. **c-d,** Kaplan-Meier curves of 50 *virtual* patients that receive immunotherapy under NS (**c**) and NFDS (**d**) with mutation rate of $\mu = 5.5$. **e-f,** Kaplan-Meier curves of 50 *virtual* patients that receive immunotherapy under NS (**e**) and NFDS (**f**) with mutation rate of $\mu = 6$.

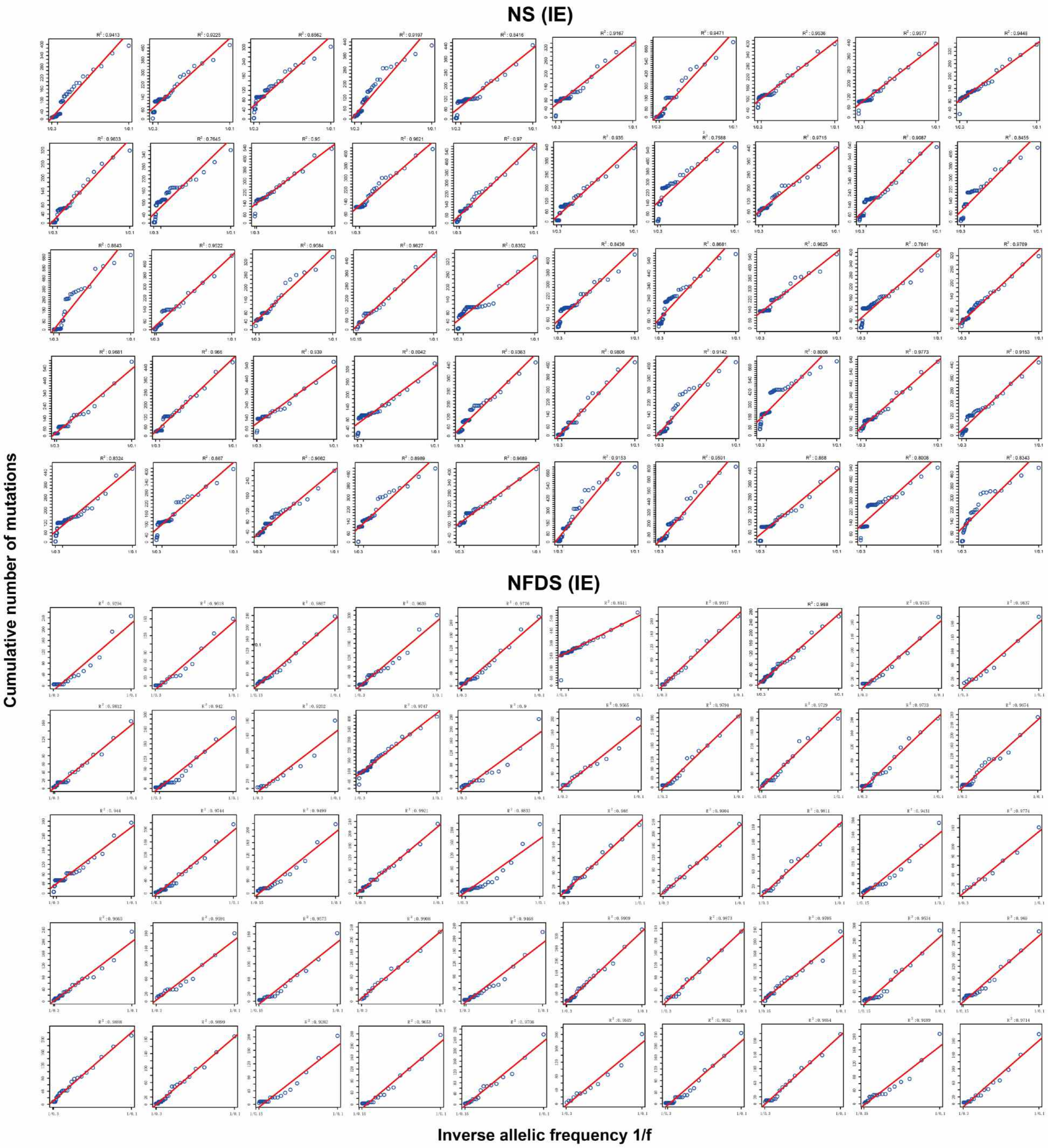

Supplementary Fig. 21. Visualized cumulative distribution of CCF and $1/f$ model fitting for simulation data.

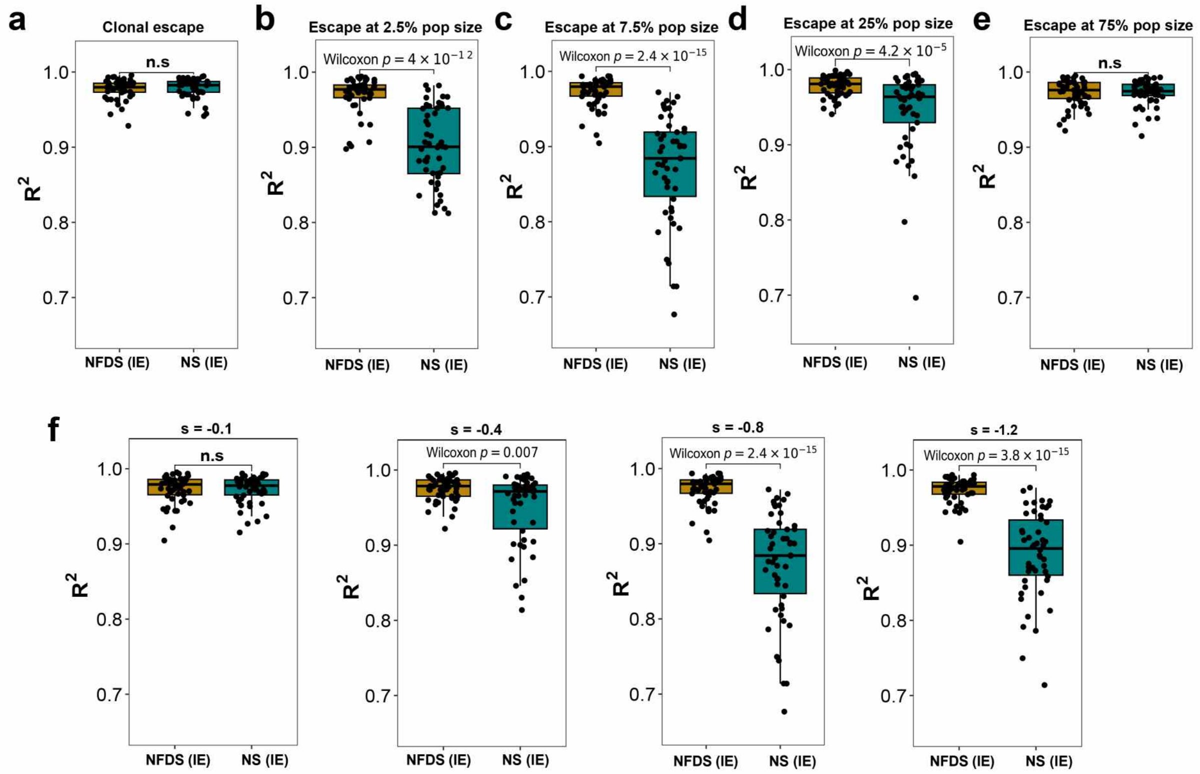

Supplementary Fig. 22. Identification of neutrality for *virtual* tumors with IE introduced at fixed points. a, Identification of neutrality with clonal escape. **b-e,** Identification of neutrality with subclonal immune escape introduced when *virtual* tumors reached population sizes of 2.5%, 7.5%, 25% and 75% of the predefined final population size, respectively. **f,** Identification of neutrality with subclonal immune escape introduced at 7.5% pop size at varying s. The R^2 represents degree of fitting to power-law distribution. p values, one-sided Wilcoxon rank-sum test.

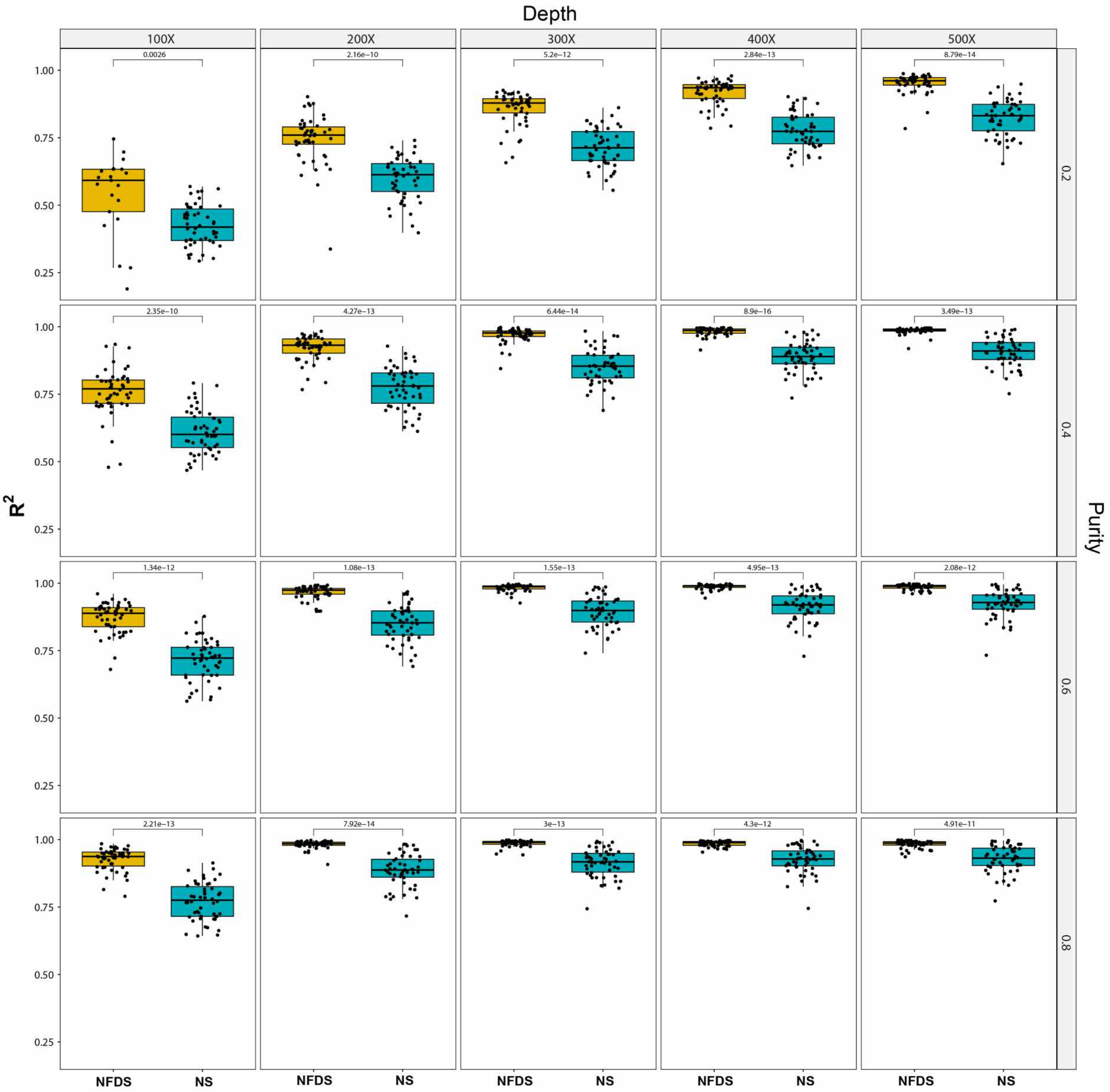

Supplementary Fig. 23. Identification of neutrality for simulated tumors. Identification of neutrality with varying sequencing depths and purities in simulations. Simulated depths range from 100x to 500x. Simulated purities range from 0.2 to 0.8. Box plots show median, quartiles (boxes) and range (whiskers). p values, one-sided Wilcoxon rank-sum test.

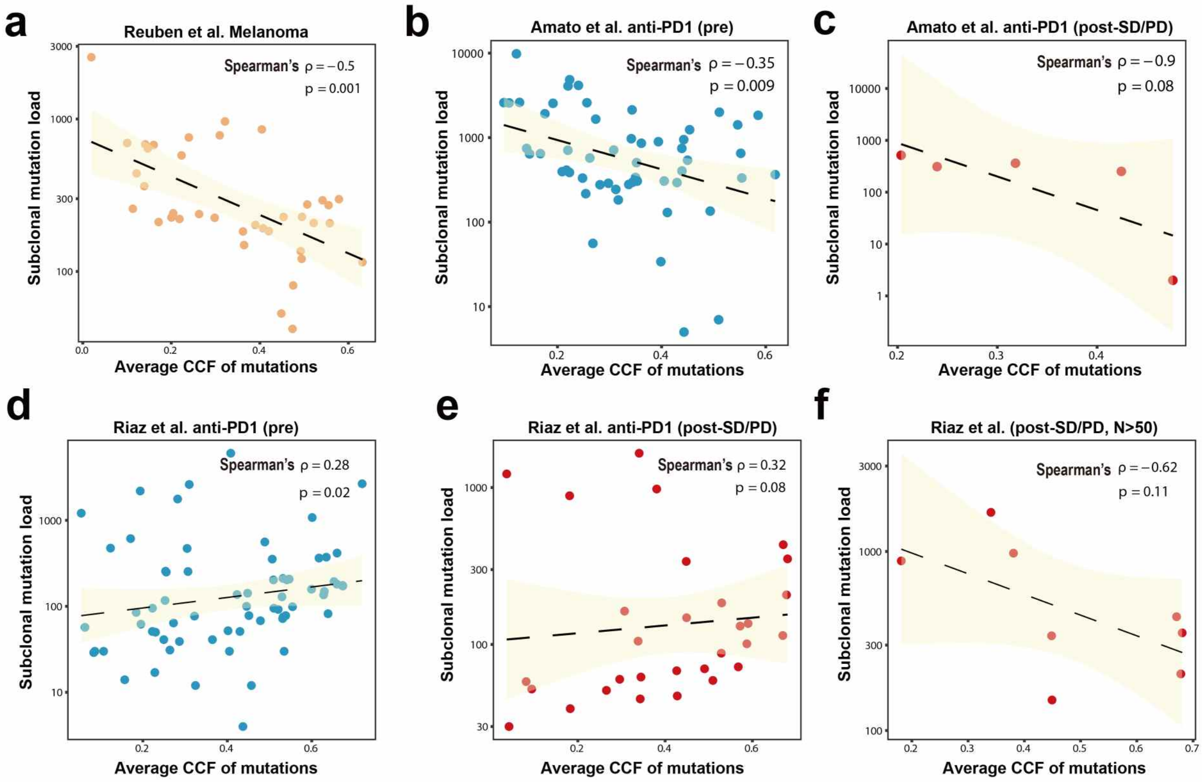

Supplementary Fig. 24. Correlation analysis for patient samples. a-f, Correlation analysis between the average CCF and number of subclonal mutations for samples (n = 38) from Reuben et al. (**a**), pre-therapy samples (n = 54) from Amato et al. (**b**), post-therapy SD/PD samples (n = 5) from Amato et al. (**c**), pre-therapy samples (n = 67) from Riaz et al. (**d**), post-therapy SD/PD samples (n = 30) from Riaz et al. (**e**), and samples (n = 8) with high subclonal neoantigen load (more than 50) from Riaz et al.(**f**). The line indicates the linear regression and the shading indicates the 95% CI of the regression.

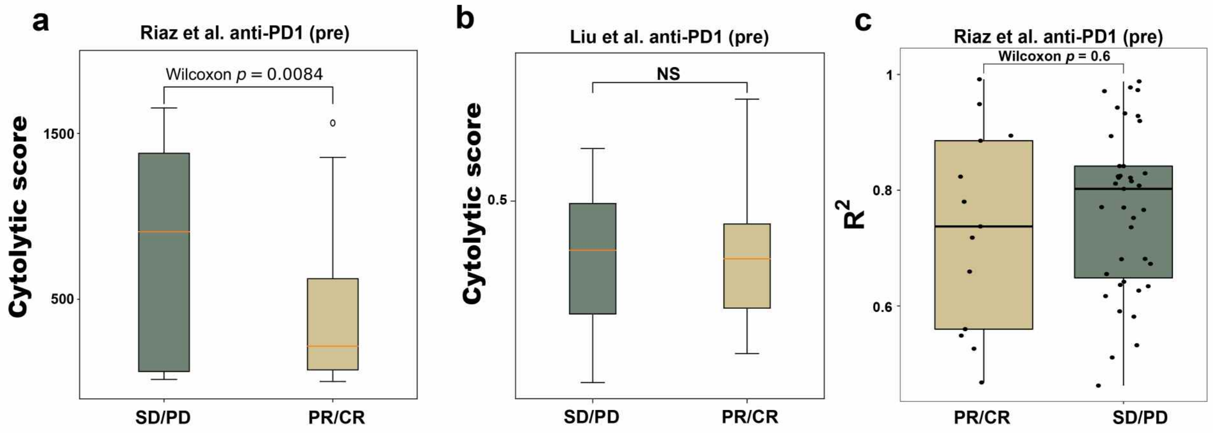

Supplementary Fig. 25. Immune infiltration in patients. a-b, Box plots showing the cytolytic score of responders (PR/CR) and non-responders (SD/PD) from Riaz et al. (n = 39 for left box and n = 13 for right box) (**a**) and Liu et al. (n = 36 for left box and n = 32 for right box) (**b**), respectively. Box plots show median, quartiles (boxes) and range (whiskers). **c,** Power-law model of neutrality fitting for responders (PR/CR, n = 13) vs non-responders (SD/PD, n = 39) from Riaz et al.' s cohort. p values, one-sided Wilcoxon rank-sum test.

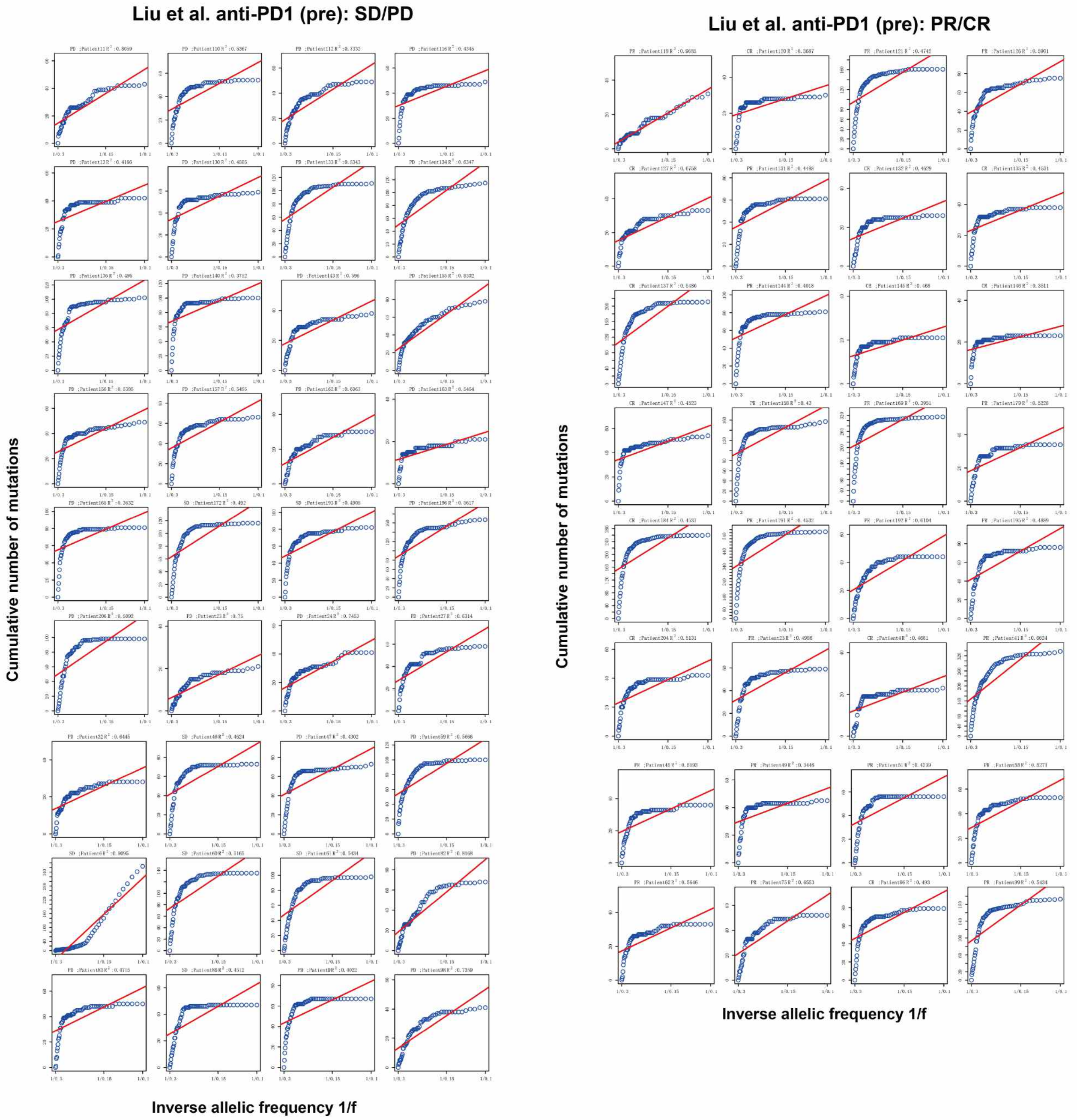

Supplementary Fig. 26. Visualized cumulative distribution of CCF and $1/f$ model fitting for samples from Liu et al.

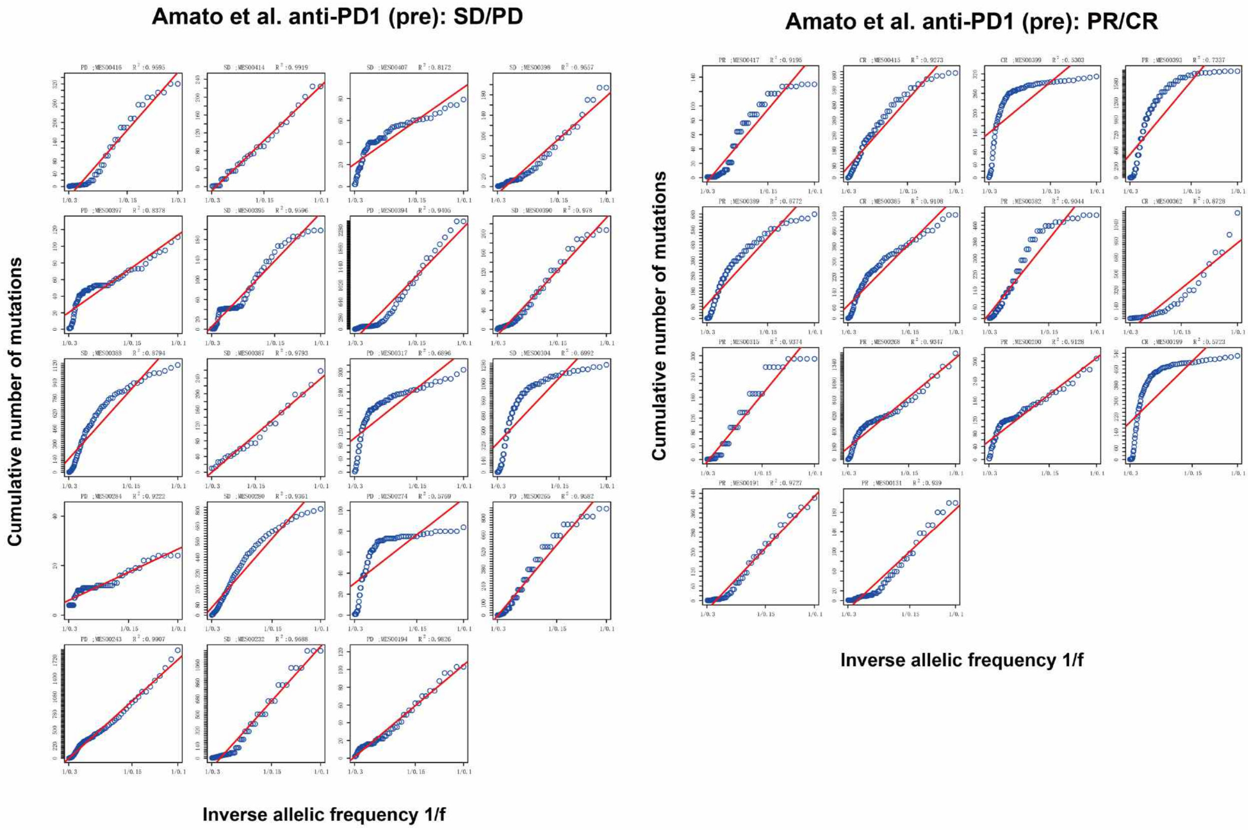

Supplementary Fig. 27. Visualized cumulative distribution of CCF and $1/f$ model fitting for samples from Amato et al.

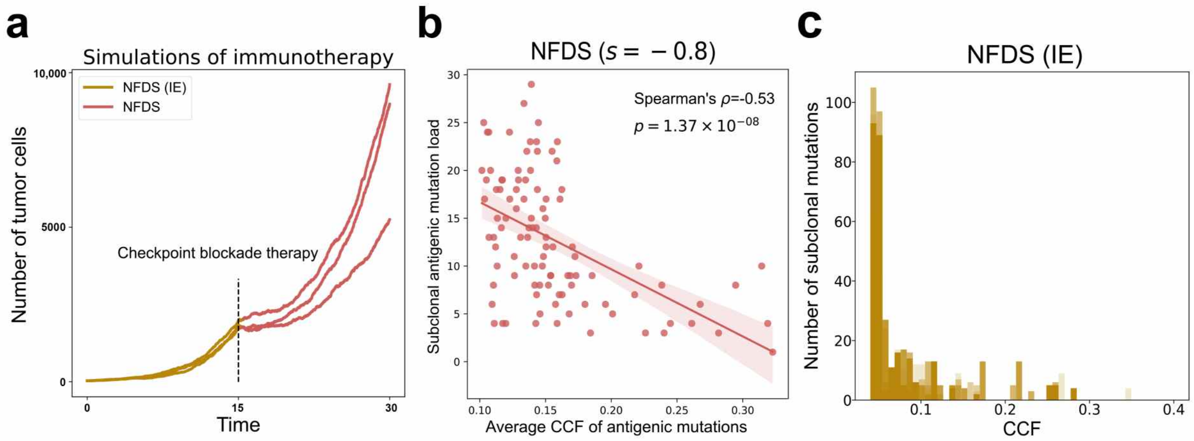

Supplementary Fig. 28. Exploratory simulations of revised NFDS. a, Growth curves of 20 simulated tumors under NS and NFDS ($\mu = 5.5$), respectively. **b,** Correlation analysis between average CCF and subclonal antigenic mutation load of 100 simulated tumors undergoing NFDS. Only neoantigens with CCF > 0.1 are used. The line indicates the linear regression and the shading indicates the 95% CI of the regression. **c,** CCF distributions of all mutations (including neutral passengers) of five simulated tumors under immune escape mode of NFDS.

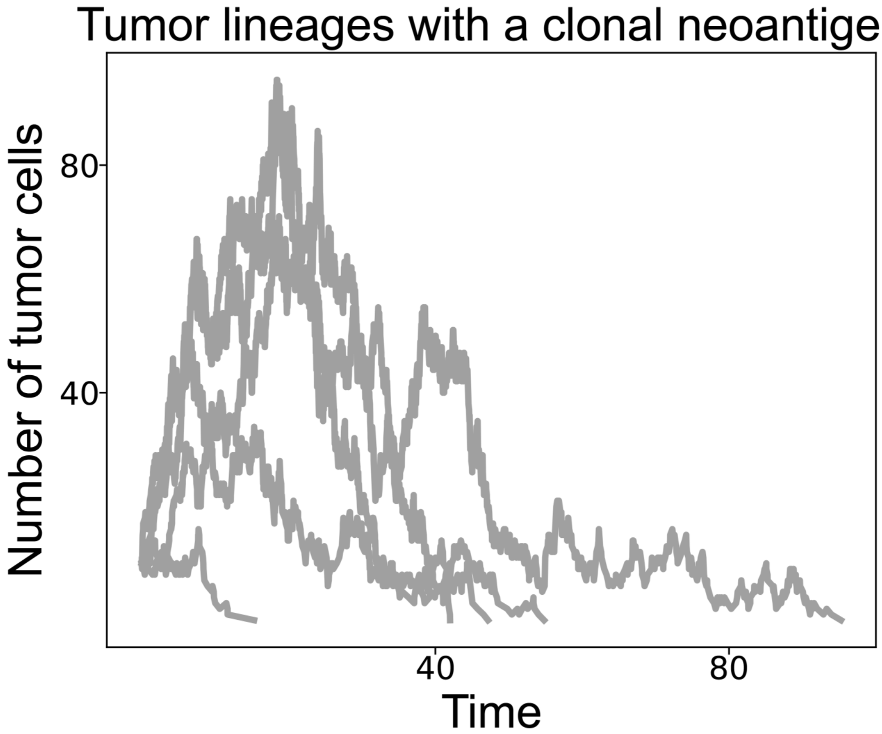

Supplementary Fig. 29. Tumor lineages with a clonal neoantigen. Five simulated tumors that each contains one clonal antigenic mutation with antigenicity score equals to 0.2 and mutation rate of $\mu = 5$ at selective intensity $s = -0.8$.

2 Supplementary Note

2.1 The ODE model

To gain deeper understanding of the simulation results, we employed an ordinary differential equation (ODE) model. This model was designed to capture the underlying mechanisms of tumor-immune interactions, drawing inspiration from the classical predator-prey model[1-3]. Since our focus is on hypermutable tumors and neoantigens, we have excluded the transition process of neutral tumor cells to immunogenic cells. To describe the early growth dynamics of an immunogenic tumor lineage i, denoted as I_i, we can use the following equations:

$$\frac{dI_i}{dt} = bI_i - ksA_{c_i}I_i, \tag{1}$$

where b and A_{c_i} represent the birth rate and antigenicity of the lineage respectively. In order to account for the frequency-dependent mechanism, both negative selection (s) and the antigenicity (A_{c_i}) of the tumor lineage were incorporated in the model. The model describes the exponential growth of tumors in the early stages of tumor progression, without considering size-related effects such as carrying capacity and Michaelis-Menten parameters[4]. This allows us to focus specifically on the initial phases of tumor development. To capture the relationship between the antigenicity of a tumor lineage and the antigenic mutation load, we made the assumption of a linear correlation. Specifically, we observed that higher neoantigen numbers were associated with an increased likelihood of presenting immunogenic cells within the lineage. Additionally, by incorporating the concept of frequency-dependent selection and drawing insights from previous studies[2, 5], we formulated the negative selection (s) as $\gamma_i \cdot s_I$ for each immunogenic tumor lineage. Similarly, we represented the antigenicity (A_{c_i}) as the fraction of all antigenic mutation load (L_i) in the lineage, denoted as $l \cdot L_i$ where l is a positive coefficient. Here, γ_i denoted the average clonal fraction of antigenic mutations within lineage i, while s_I represented the intensity of negative selection.

The dynamics of the immunogenic tumor population can be described by the following equation, which considers the antigenicity of each lineage within the tumor:

$$\sum_i^n \frac{dI_i}{dt} = \sum_i^n I_i \left(b - kA_{c_i}s_I\gamma_i\right), \tag{2}$$

where I_i represents the population of the i-th immunogenic lineage of the tumor and n represents the total number of subclones. In growing tumors we have

$$\sum_i^n \frac{dI_i}{dt} = \sum_i^n I_i \left(b - kA_{c_i}s_I\gamma_i\right) > 0. \tag{3}$$

Assuming that each tumor lineage evolves at same mutation rate, we have $I_i \approx I_j$ and set $\gamma_i \approx \gamma_j \approx \gamma$ $(i \neq j)$, where γ represents the average CCF of all antigenic mutations in the tumor, which leads to

$$\frac{b}{kl \cdot s_I}\frac{n}{\gamma} > \sum_i L_i. \tag{4}$$

The inequality above emphasizes the connection between the antigenic mutation load $\sum_i L_i$ on the right-hand side and $\frac{n}{\gamma}$ on the left-hand side. Drawing inferences from inequality (4), we can conclude that higher levels of intratumoral neoantigen heterogeneity enable a greater capacity for larger neoantigen loads in growing tumors. This implies

that tumors with increased intratumoral neoantigen heterogeneity have the potential to accommodate a higher burden of neoantigens as they advance in progression.

We then modeled subclonal immune escape as follows:

$$\begin{aligned} \frac{dI}{dt} &= b \cdot I - ksA_cI - p_eI, \\ \frac{dE}{dt} &= b \cdot E + 2p_eI, \end{aligned} \tag{5}$$

where I represents the population of immunogenic cells, therefore we have $I = \sum_i I_i$, $A_c = \sum_i A_{c_i}$. Additionally, we introduce E to represent the population of immune escaped cells, and p_e denotes the probability of immune escape. Based on equation (5), we can infer the following relationship in growing tumors:

$$\frac{dT}{dt} = b \cdot I - ksI \sum_i l \cdot L_i + b \cdot E + p_eI > 0, \tag{6}$$

where T represents total number of tumor cells. Under the assumption that $T > 0$ and note that $I + E = T$, we have $\frac{I}{T} \leq 1$ and these analyses finally lead to the following inequality:

$$\sum_i L_i < \frac{b + p_e}{kls\frac{I}{T}}. \tag{7}$$

We can deduce from equation (7) that a higher probability of subclonal immune escape allows a tumor to accommodate a larger number of antigenic mutations. Consequently, early subclonal immune escape ensures that the tumor presents a greater number of antigenic mutations with higher clonal fraction (CCF), resulting in an increased antigenic mutation load.

The mathematical analyses presented above highlights different correlation patterns between average CCF and antigenic mutation load in the frequency-dependent selection model and the immune escape model. In tumors subjected to frequency-dependent selection, there exists a negative correlation between the average CCF and antigenic mutation load. However, in tumors exhibiting immune escape mechanisms alone, the average CCF is positively correlated with the antigenic mutation load.

Supplementary References

[1] Eladdadi, A., Kim, P., Mallet, D.: Mathematical Models of Tumor-immune System Dynamics vol. 107. Springer, New York (2014)

[2] Aguadé-Gorgorió, G., Solé, R.: Tumour neoantigen heterogeneity thresholds provide a time window for combination immunotherapy. Journal of the Royal Society Interface **17**(171), 20200736 (2020)

[3] Bellomo, N., Preziosi, L.: Modelling and mathematical problems related to tumor evolution and its interaction with the immune system. Mathematical and Computer Modelling **32**(3-4), 413–452 (2000)

[4] Łuksza, M., *et al.*: A neoantigen fitness model predicts tumour response to checkpoint blockade therapy. Nature **551**(7681), 517–520 (2017)

[5] Johnson, K.A., Goody, R.S.: The original michaelis constant: translation of the 1913 michaelis–menten paper. Biochemistry **50**(39), 8264–8269 (2011)
